# 2-{2-[4-(Dimethyl­amino)­phen­yl]diazen-1-ium-1-yl}pyridinium tetra­chlorido­zincate

**DOI:** 10.1107/S1600536812023689

**Published:** 2012-05-31

**Authors:** Nararak Leesakul, Wassana Runrueng, Saowanit Saithong, Chaveng Pakawatchai

**Affiliations:** a Department of Chemistry and Center for Innovation in Chemistry, Faculty of Science, Prince of Songkla University, Hat Yai, Songkhla 90112, Thailand

## Abstract

The title compound, (C_13_H_16_N_4_)[ZnCl_4_], consists of a tetra­hedral [ZnCl_4_]^2−^ anion and a 2-{2-[4-(dimethyl­amino)­phen­yl]diazen-1-ium-1-yl}pyridinium dication. The pyridinium-N atom is *syn* to the azo bond which allows for the formation of an intramolecular N—H⋯N hydrogen bond. In the crystal, the cation and anion are held together by N—H⋯Cl hydrogen-bond inter­actions involving the pyridinium and diazen-1-ium N atoms. π–π stacking inter­actions occur between the pyridine and benzene rings of adjacent cations [centroid–centroid distances = 3.6270 (18) and 3.8685 (18) Å]; the stacks are parallel to the *a* axis.

## Related literature

For background to azo complexes, see: Chand *et al.* (2003 ▶); Das *et al.* (2006 ▶); Arslan (2007 ▶). For structures of related azoimine compounds and complexes, see: Panneerselvam *et al.* (2000 ▶); Leesakul *et al.* (2010 ▶, 2011 ▶). For structure of tetra­chloro­zincate (II), see: Harrison (2005 ▶); Valdés-Martínez *et al.* (2005 ▶); Bringley & Rajeswaran (2006 ▶); Xu *et al.* (2005 ▶).
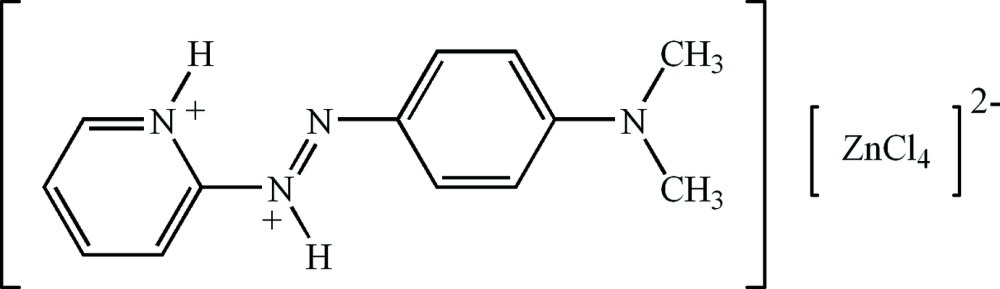


## Experimental

### 

#### Crystal data

(C_13_H_16_N_4_)[ZnCl_4_]
*M* *_r_* = 435.47
Monoclinic, 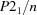

*a* = 7.4556 (4) Å
*b* = 21.4126 (10) Å
*c* = 11.1924 (5) Å
β = 99.883 (1)°
*V* = 1760.28 (15) Å^3^
*Z* = 4
Mo *K*α radiation
μ = 2.00 mm^−1^
*T* = 293 K
0.18 × 0.17 × 0.04 mm

#### Data collection

Bruker APEX CCD area-detector diffractometer
Absorption correction: multi-scan (*SADABS*; Bruker, 2003) ▶ *T*_min_ = 0.699, *T*_max_ = 0.929
16401 measured reflections
3094 independent reflections
2687 reflections with *I* > 2σ(*I*)
*R*_int_ = 0.034

#### Refinement

*R*[*F* ^2^ > 2σ(*F* ^2^)] = 0.035
*wR*(*F* ^2^) = 0.082
*S* = 1.10
3094 reflections
209 parameters
2 restraints
H atoms treated by a mixture of independent and constrained refinement
Δρ_max_ = 0.36 e Å^−3^
Δρ_min_ = −0.22 e Å^−3^

### 

Data collection: *SMART* (Bruker, 1998 ▶); cell refinement: *SAINT* (Bruker, 2003 ▶); data reduction: *SAINT*; program(s) used to solve structure: *SHELXS97* (Sheldrick, 2008 ▶); program(s) used to refine structure: *SHELXL97* (Sheldrick, 2008 ▶); molecular graphics: *Mercury* (Macrea *et al.*, 2008 ▶); software used to prepare material for publication: *SHELXL97*, *WinGX* (Farrugia, 1999 ▶) and *publCIF* (Westrip, 2010 ▶).

## Supplementary Material

Crystal structure: contains datablock(s) I, global. DOI: 10.1107/S1600536812023689/qm2069sup1.cif

Structure factors: contains datablock(s) I. DOI: 10.1107/S1600536812023689/qm2069Isup2.hkl

Additional supplementary materials:  crystallographic information; 3D view; checkCIF report

## Figures and Tables

**Table 1:** Hydrogen-bond geometry (Å, °)

*D*—H⋯*A*	*D*—H	H⋯*A*	*D*⋯*A*	*D*—H⋯*A*
N1—H1*A*⋯N3	0.89 (2)	2.21 (3)	2.595 (3)	106 (2)
N2—H2*A*⋯Cl3^i^	0.90 (2)	2.45 (2)	3.322 (3)	165 (3)
N1—H1*A*⋯Cl1^ii^	0.89 (2)	2.37 (2)	3.173 (3)	150 (3)

Symmetry codes: (i) 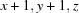; (ii) 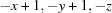.

**Fig. 1.**
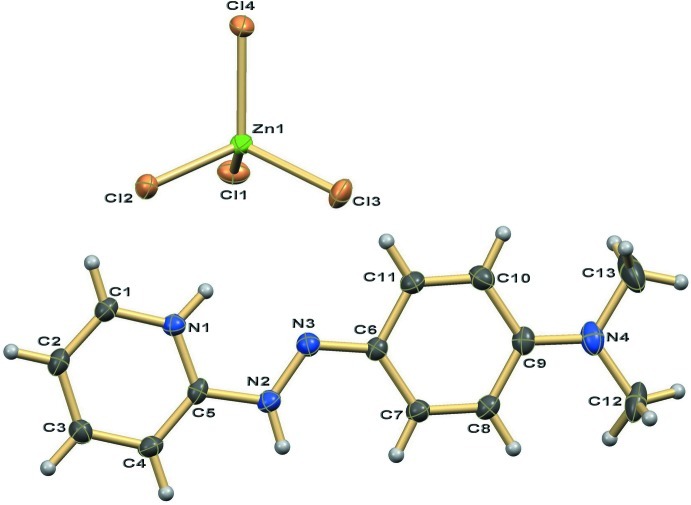
Molecular structure of [(H2dmazpy)2+(ZnCl4)2-] with thermal ellipsoids plotted at the 50% probability level.

**Fig. 2.**
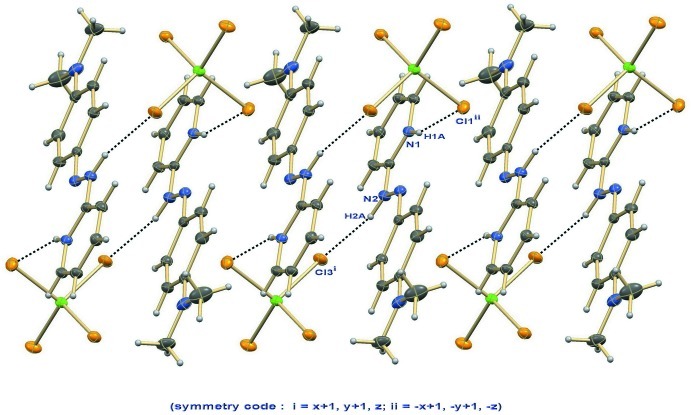
Hydrogen bonding interactions of [(H2dmazpy)2+(ZnCl4)2-].

**Fig. 3.**
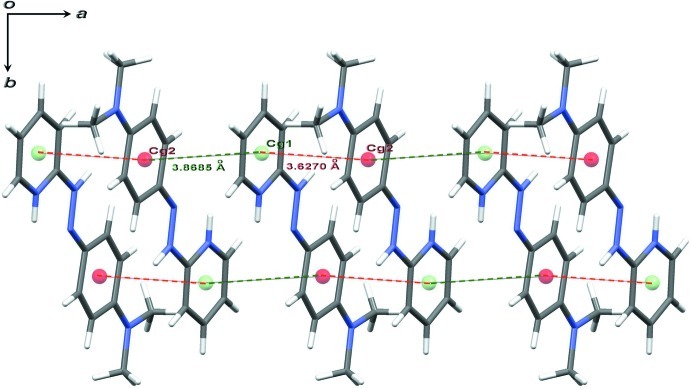
The π···π stacking between phenyl and pyriding rings of (H2dmazpy)2+.

## supplementary crystallographic information

### Comment

The molecule existing azoimine,—N═N—C═N—, functional group is known to have strong π-acidity and to efficiently stabilize transition metal ions (Arslan, 2007). However, the chemistry of zinc with the azoimine moiety has remained less explored. There has been a substantial investigation of the chemistry of Zn(II) complexes of N-donor heterocycles (Das *et al.*, 2006 and Chand *et al.*, 2003) as possible optical materials. In an effort towards the design of azoimine containing ligands we recently reported the crystal structures of *N,N*-dimethyl-4-[(2-pyridyldiazenyl]aniline (dmazpy) ligand (Leesakul *et al.*, 2010) and the distorted tetrahedral geometry of a neutral Zn(II) coordination compound with *N,N*-diethyl-4-[(2-pyridyldiazenyl]aniline (deazpy) ligand, [ZnCl_2_(Cl_15_H_18_N_4_)_2_] or [ZnCl_2_(deazpy)_2_] (Leesakul *et al.*, 2011).

Herein, we have a similar Zn(II) complex synthesis pathway with differences in the ligand type and the purification method, the ionic structure of *N,N*-dimethyl-4-[(2-pyridyliumdiazenylium]aniline tetrachlorozincate(II), (C_13_H_16_N_4_)[ZnCl_4_] or (H_2_dmazpy).[ZnCl_4_], was obtained (Scheme I). The title compound, Fig.1, contains an alternating part of an anionic tetrahedral [ZnCl_4_]^2-^ and pyridyliumazenylium cation, (H_2_dmazpy)^2+^. The structure is commonly observed in other related compounds *e.g.* 4-*tert*-Butyl-2,6-bis[imidazolium-1-yl)methyl]phenol tetrachloro zincate(II) (Xu *et al.*, 2005), *bis*(quinolinium)tetrachlorozincate dehydrate (Valdés-Martínez *et al.*, 2005) and *p*-Phenylenediammonium tetrachlorozincate (II) (Bringley *et al.*, 2006).

The mean value of the Zn—Cl bond distance of ZnCl_4_^2-^ anion is 2.266 (8) Å which is generally observed [2.268 (4) Å] (Harrison, 2005). The Cl—Zn—Cl bond angles in Fig. 1 deviate from 109.5° only slightly [108.19 (3)°-111.53 (3)°]. The cationic species (H_2_dmazpy)^2+^ is doubly protonated on the pyridine N1 and azo N2 due to their higher electron density. The N atom of the pyridine ring of the cation adopts a *cis-*orientation with respect to the azo moiety (—N2═ N3—) which is in contrast to the *trans*-geometry of the free dmazpy ligand (Leesakul *et al.*, 2010). However, it is similar to an observation in a related crystal structure of 2-(4-hydroxyphenylazo)pyridine (3:1) tetrafluoroborate (Panneerselvam *et al.*, 2000). Nevertheless, only single a protonation on the azo N3 was found. In the title compound, the dihedral angle of mean plane of pyridine-azo-phenyl rings is 2.38 (15)°. The N═N distance of the (H_2_dmazpy)^2+^ is 1.313 (3) Å which is obviously longer than that of the free dmazpy ligand, 1.257 (16) Å. It may be caused by the protonation on the N atom of azo group which decreases the azo bond strength in comparison with the related free dmazpy ligand.

It is worth noting that the two Cl atoms of [Zn—Cl_4_]^2-^ are linked to the protonated pyridine H1A and the protonated N-azo H2A *via* H-bonding, with an N1···Cl1 distance of 3.173 (3) Å and an N2···Cl3 distance of 3.322 (3) Å, respectively. (see Fig. 2 and Table 1). In the crystal structure, the intermolecular π*-*π stacking interactions (Fig. 3) occur between adjacent pyridine (*Cg*1) and phenyl rings (*Cg*2). The centroid-centroid distances, *Cg*1···*Cg*2, in the stacks which are parallel to the *a* axis are 3.6270 (18) Å and 3.8685 (18) Å respectively.

### Experimental

The *N,N*-dimethyl-4-[2-(pyridyl)diazenyl]aniline) compound was prepared by the method from our previous publication (Leesakul *et al.*, 2010). An acetonitrile solution (20 ml) of The *N,N*-dimethyl-4-[2-(pyridyl)diazenyl]aniline) (0.14 g, 0.6 mmol) and ZnCl_2_ (0.04 g, 0.3 mmol) was refluxed for 4 h. The warm reaction mixture at 75°C was filtered. The filtrate was extracted with water and CH_2_Cl_2_ (1:1) in order to remove the excess *N,N*-dimethyl-4-[2-(pyridyl)diazenyl]aniline) compound by CH_2_Cl_2_. The filtrate was evaporated and washed the precipitate with CH_2_Cl_2_ and diethylether, respectively for twice times. The dark red solids were recrystallized with dichloromethane and methanol (1:2) at room temperature for a week. The redbrown crystals were obtained (yield 37%, 0.05 g).

### Refinement

The structure was solved by direct methods refined by a full-matrix least-squares procedure based on *F^2^*. All hydrogen atoms on C atoms were constrained, C—H = 0.9300 Å with *U*_iso_(H) = 1.2*U*_eq_(C) for C-*sp*^2^ atoms and C—H = 0.9600 Å with *U*_iso_(H) = 1.5*U*_eq_(C) for C-*sp*^3^ atoms of methyl groups, respectively. The hydrongen atoms of N atoms are located in a difference map and restrained, N—H = 0.89 Å with *U*_iso_(H) = 1.2*U*_eq_(N).

### Crystal data

**Table:**

(C_13_H_16_N_4_)[ZnCl_4_]	*F*(000) = 880
*M**_r_* = 435.47	*D*_x_ = 1.643 Mg m^−^^3^
Monoclinic, *P*2_1_/*n*	Mo *K*α radiation, λ = 0.71073 Å
Hall symbol: -P 2yn	Cell parameters from 3620 reflections
*a* = 7.4556 (4) Å	θ = 2.7–23.6°
*b* = 21.4126 (10) Å	µ = 2.00 mm^−^^1^
*c* = 11.1924 (5) Å	*T* = 293 K
β = 99.883 (1)°	Block, redbrown
*V* = 1760.28 (15) Å^3^	0.18 × 0.17 × 0.04 mm
*Z* = 4	

### Data collection

**Table:**

Bruker APEX CCD area-detector diffractometer	3094 independent reflections
Radiation source: fine-focus sealed tube	2687 reflections with *I* > 2σ(*I*)
Graphite monochromator	*R*_int_ = 0.034
Frames, each covering 0.3 ° in ω scans	θ_max_ = 25.0°, θ_min_ = 1.9°
Absorption correction: multi-scan (*SADABS*; Bruker, 2003)	*h* = −8→8
*T*_min_ = 0.699, *T*_max_ = 0.929	*k* = −25→25
16401 measured reflections	*l* = −13→13

### Refinement

**Table:**

Refinement on *F*^2^	Primary atom site location: structure-invariant direct methods
Least-squares matrix: full	Secondary atom site location: difference Fourier map
*R*[*F*^2^ > 2σ(*F*^2^)] = 0.035	Hydrogen site location: inferred from neighbouring sites
*wR*(*F*^2^) = 0.082	H atoms treated by a mixture of independent and constrained refinement
*S* = 1.10	*w* = 1/[σ^2^(*F*_o_^2^) + (0.038*P*)^2^ + 0.8053*P*] where *P* = (*F*_o_^2^ + 2*F*_c_^2^)/3
3094 reflections	(Δ/σ)_max_ = 0.008
209 parameters	Δρ_max_ = 0.36 e Å^−^^3^
2 restraints	Δρ_min_ = −0.22 e Å^−^^3^

### Special details

**Table:**

Geometry. All e.s.d.'s (except the e.s.d. in the dihedral angle between two l.s. planes) are estimated using the full covariance matrix. The cell e.s.d.'s are taken into account individually in the estimation of e.s.d.'s in distances, angles and torsion angles; correlations between e.s.d.'s in cell parameters are only used when they are defined by crystal symmetry. An approximate (isotropic) treatment of cell e.s.d.'s is used for estimating e.s.d.'s involving l.s. planes.
Refinement. Refinement of *F*^2^ against all reflections. The weighted *R*-factor *wR* and goodness of fit *S* are based on *F*^2^, conventional *R*-factors *R* are based on *F*, with *F* set to zero for negative *F*^2^. The threshold expression of *F*^2^ > 2*σ* (*F*^2^) is used only for calculating *R*-factors(gt) *etc*. and is not relevant to the choice of reflections for refinement. *R*-factors based on *F*^2^ are statistically about twice as large as those based on *F*, and *R*- factors based on all data will be even larger.

### Fractional atomic coordinates and isotropic or equivalent isotropic displacement parameters (Å^2^)

**Table:**

	*x*	*y*	*z*	*U*_iso_*/*U*_eq_	
H2A	0.850 (5)	1.0694 (15)	0.076 (3)	0.078 (12)*	
H1A	0.605 (4)	0.9976 (9)	−0.157 (3)	0.048 (9)*	
Zn1	0.31545 (5)	0.131012 (15)	0.31166 (3)	0.03901 (12)	
Cl1	0.53360 (12)	0.10101 (4)	0.20333 (8)	0.0551 (2)	
Cl2	0.27049 (11)	0.05405 (4)	0.44071 (7)	0.0493 (2)	
Cl3	0.05128 (12)	0.14947 (4)	0.18109 (7)	0.0590 (2)	
Cl4	0.41086 (13)	0.21647 (3)	0.42151 (7)	0.0553 (2)	
N1	0.6143 (3)	1.03805 (11)	−0.1729 (2)	0.0392 (6)	
C1	0.5358 (4)	1.06175 (15)	−0.2791 (3)	0.0428 (7)	
H1	0.4752	1.0356	−0.3392	0.051*	
C2	0.5442 (4)	1.12383 (15)	−0.2993 (3)	0.0467 (8)	
H2	0.4896	1.1406	−0.3733	0.056*	
C3	0.6348 (4)	1.16232 (15)	−0.2090 (3)	0.0465 (8)	
H3	0.6396	1.2052	−0.2219	0.056*	
C4	0.7171 (4)	1.13728 (14)	−0.1010 (3)	0.0448 (7)	
H4	0.7791	1.1626	−0.0400	0.054*	
C5	0.7062 (4)	1.07333 (14)	−0.0843 (2)	0.0383 (7)	
N2	0.7867 (4)	1.04376 (12)	0.0205 (2)	0.0426 (6)	
N3	0.7675 (3)	0.98290 (11)	0.0249 (2)	0.0416 (6)	
C6	0.8345 (4)	0.95095 (13)	0.1231 (2)	0.0361 (6)	
C7	0.9312 (4)	0.97440 (14)	0.2352 (2)	0.0423 (7)	
H7	0.9528	1.0171	0.2442	0.051*	
C8	0.9916 (4)	0.93585 (15)	0.3278 (3)	0.0452 (7)	
H8	1.0536	0.9524	0.4000	0.054*	
C9	0.9629 (4)	0.86986 (15)	0.3183 (3)	0.0430 (7)	
C10	0.8646 (5)	0.84604 (15)	0.2046 (3)	0.0500 (8)	
H10	0.8424	0.8034	0.1953	0.060*	
C11	0.8059 (4)	0.88491 (14)	0.1132 (3)	0.0451 (7)	
H11	0.7444	0.8686	0.0406	0.054*	
N4	1.0275 (4)	0.83274 (14)	0.4091 (2)	0.0580 (8)	
C12	1.1324 (6)	0.8561 (2)	0.5244 (3)	0.0789 (13)	
H12A	1.2590	0.8583	0.5181	0.118*	
H12B	1.1169	0.8281	0.5890	0.118*	
H12C	1.0894	0.8969	0.5411	0.118*	
C13	1.0056 (8)	0.7651 (2)	0.4030 (4)	0.1017 (17)	
H13A	1.0638	0.7489	0.3394	0.152*	
H13B	0.8784	0.7549	0.3867	0.152*	
H13C	1.0604	0.7470	0.4790	0.152*	

### Atomic displacement parameters (Å^2^)

**Table:**

	*U*^11^	*U*^22^	*U*^33^	*U*^12^	*U*^13^	*U*^23^
Zn1	0.0420 (2)	0.0397 (2)	0.0336 (2)	0.00047 (15)	0.00148 (14)	−0.00352 (14)
Cl1	0.0547 (5)	0.0528 (5)	0.0623 (5)	−0.0067 (4)	0.0225 (4)	−0.0154 (4)
Cl2	0.0582 (5)	0.0471 (4)	0.0393 (4)	−0.0050 (4)	−0.0010 (3)	0.0049 (3)
Cl3	0.0521 (5)	0.0764 (6)	0.0420 (4)	−0.0008 (4)	−0.0100 (4)	0.0068 (4)
Cl4	0.0763 (6)	0.0344 (4)	0.0496 (4)	0.0049 (4)	−0.0055 (4)	−0.0063 (3)
N1	0.0417 (14)	0.0384 (14)	0.0374 (13)	0.0002 (11)	0.0067 (11)	−0.0037 (11)
C1	0.0406 (17)	0.0515 (18)	0.0347 (15)	0.0010 (14)	0.0015 (13)	−0.0043 (14)
C2	0.0456 (18)	0.058 (2)	0.0344 (16)	0.0057 (15)	0.0001 (14)	0.0040 (14)
C3	0.0498 (19)	0.0415 (17)	0.0477 (18)	−0.0028 (14)	0.0068 (15)	0.0051 (14)
C4	0.0456 (18)	0.0466 (18)	0.0398 (17)	−0.0047 (14)	0.0008 (14)	−0.0094 (14)
C5	0.0327 (15)	0.0524 (18)	0.0297 (14)	0.0057 (13)	0.0054 (12)	0.0029 (13)
N2	0.0463 (15)	0.0449 (15)	0.0353 (14)	0.0000 (12)	0.0033 (11)	−0.0046 (11)
N3	0.0438 (15)	0.0418 (14)	0.0394 (13)	0.0019 (11)	0.0084 (11)	−0.0007 (11)
C6	0.0349 (15)	0.0420 (16)	0.0326 (14)	0.0010 (12)	0.0092 (12)	−0.0003 (12)
C7	0.0467 (18)	0.0425 (16)	0.0374 (16)	−0.0050 (14)	0.0064 (13)	−0.0020 (13)
C8	0.0451 (18)	0.0538 (19)	0.0355 (16)	−0.0011 (15)	0.0034 (14)	−0.0026 (14)
C9	0.0403 (17)	0.0527 (18)	0.0387 (16)	0.0078 (14)	0.0141 (14)	0.0064 (14)
C10	0.061 (2)	0.0394 (17)	0.0510 (19)	−0.0001 (15)	0.0144 (17)	−0.0018 (15)
C11	0.0525 (19)	0.0450 (17)	0.0367 (16)	−0.0015 (14)	0.0048 (14)	−0.0056 (14)
N4	0.0671 (19)	0.0623 (18)	0.0488 (16)	0.0211 (15)	0.0216 (15)	0.0134 (14)
C12	0.086 (3)	0.107 (3)	0.042 (2)	0.045 (3)	0.004 (2)	0.017 (2)
C13	0.166 (5)	0.062 (3)	0.081 (3)	0.032 (3)	0.033 (3)	0.031 (2)

### Geometric parameters (Å, º)

**Table:**

Zn1—Cl4	2.2508 (8)	C6—C7	1.427 (4)
Zn1—Cl2	2.2539 (8)	C6—C11	1.431 (4)
Zn1—Cl3	2.2766 (8)	C7—C8	1.341 (4)
Zn1—Cl1	2.2815 (9)	C7—H7	0.9300
N1—C1	1.332 (4)	C8—C9	1.430 (4)
N1—C5	1.338 (4)	C8—H8	0.9300
N1—H1A	0.889 (17)	C9—N4	1.314 (4)
C1—C2	1.352 (4)	C9—C10	1.448 (4)
C1—H1	0.9300	C10—C11	1.333 (4)
C2—C3	1.387 (4)	C10—H10	0.9300
C2—H2	0.9300	C11—H11	0.9300
C3—C4	1.368 (4)	N4—C13	1.458 (5)
C3—H3	0.9300	N4—C12	1.476 (5)
C4—C5	1.386 (4)	C12—H12A	0.9600
C4—H4	0.9300	C12—H12B	0.9600
C5—N2	1.376 (4)	C12—H12C	0.9600
N2—N3	1.313 (3)	C13—H13A	0.9600
N2—H2A	0.901 (18)	C13—H13B	0.9600
N3—C6	1.318 (4)	C13—H13C	0.9600
			
Cl4—Zn1—Cl2	108.19 (3)	C8—C7—C6	121.0 (3)
Cl4—Zn1—Cl3	111.53 (3)	C8—C7—H7	119.5
Cl2—Zn1—Cl3	109.32 (3)	C6—C7—H7	119.5
Cl4—Zn1—Cl1	109.38 (4)	C7—C8—C9	121.7 (3)
Cl2—Zn1—Cl1	109.43 (3)	C7—C8—H8	119.2
Cl3—Zn1—Cl1	108.95 (4)	C9—C8—H8	119.2
C1—N1—C5	122.5 (3)	N4—C9—C8	120.7 (3)
C1—N1—H1A	121 (2)	N4—C9—C10	122.0 (3)
C5—N1—H1A	117 (2)	C8—C9—C10	117.4 (3)
N1—C1—C2	119.9 (3)	C11—C10—C9	120.3 (3)
N1—C1—H1	120.1	C11—C10—H10	119.8
C2—C1—H1	120.1	C9—C10—H10	119.8
C1—C2—C3	119.5 (3)	C10—C11—C6	122.2 (3)
C1—C2—H2	120.2	C10—C11—H11	118.9
C3—C2—H2	120.2	C6—C11—H11	118.9
C4—C3—C2	120.0 (3)	C9—N4—C13	122.8 (3)
C4—C3—H3	120.0	C9—N4—C12	122.7 (3)
C2—C3—H3	120.0	C13—N4—C12	114.5 (3)
C3—C4—C5	118.6 (3)	N4—C12—H12A	109.5
C3—C4—H4	120.7	N4—C12—H12B	109.5
C5—C4—H4	120.7	H12A—C12—H12B	109.5
N1—C5—N2	117.7 (3)	N4—C12—H12C	109.5
N1—C5—C4	119.5 (3)	H12A—C12—H12C	109.5
N2—C5—C4	122.8 (3)	H12B—C12—H12C	109.5
N3—N2—C5	117.0 (2)	N4—C13—H13A	109.5
N3—N2—H2A	129 (2)	N4—C13—H13B	109.5
C5—N2—H2A	114 (2)	H13A—C13—H13B	109.5
N2—N3—C6	121.2 (2)	N4—C13—H13C	109.5
N3—C6—C7	127.8 (3)	H13A—C13—H13C	109.5
N3—C6—C11	114.7 (3)	H13B—C13—H13C	109.5
C7—C6—C11	117.5 (3)		
			
C5—N1—C1—C2	−1.4 (5)	C11—C6—C7—C8	−0.5 (4)
N1—C1—C2—C3	0.0 (5)	C6—C7—C8—C9	0.4 (5)
C1—C2—C3—C4	1.0 (5)	C7—C8—C9—N4	178.2 (3)
C2—C3—C4—C5	−0.5 (5)	C7—C8—C9—C10	−0.4 (5)
C1—N1—C5—N2	−178.1 (3)	N4—C9—C10—C11	−177.9 (3)
C1—N1—C5—C4	1.8 (4)	C8—C9—C10—C11	0.6 (5)
C3—C4—C5—N1	−0.8 (5)	C9—C10—C11—C6	−0.8 (5)
C3—C4—C5—N2	179.1 (3)	N3—C6—C11—C10	−179.7 (3)
N1—C5—N2—N3	0.4 (4)	C7—C6—C11—C10	0.7 (5)
C4—C5—N2—N3	−179.5 (3)	C8—C9—N4—C13	−178.5 (4)
C5—N2—N3—C6	−177.6 (3)	C10—C9—N4—C13	−0.1 (5)
N2—N3—C6—C7	0.7 (5)	C8—C9—N4—C12	−0.2 (5)
N2—N3—C6—C11	−178.8 (3)	C10—C9—N4—C12	178.2 (3)
N3—C6—C7—C8	−180.0 (3)		

### Hydrogen-bond geometry (Å, º)

**Table:**

*D*—H···*A*	*D*—H	H···*A*	*D*···*A*	*D*—H···*A*
N1—H1*A*···N3	0.89 (2)	2.21 (3)	2.595 (3)	106 (2)
N2—H2*A*···Cl3^i^	0.90 (2)	2.45 (2)	3.322 (3)	165 (3)
N1—H1*A*···Cl1^ii^	0.89 (2)	2.37 (2)	3.173 (3)	150 (3)

Symmetry codes: (i) *x*+1, *y*+1, *z*; (ii) −*x*+1, −*y*+1, −*z*.
